# Olanzapine and pulmonary embolism, a rare association: a case report

**DOI:** 10.1186/1757-1626-3-36

**Published:** 2010-01-22

**Authors:** Julian FZ Maempel, Geraldine Darmanin, Kashif Naeem, Mehool Patel

**Affiliations:** 1 Department of Trauma and Orthopaedics, Frenchay Hospital, North Bristol National Health Service Trust, Bristol, UK; 2 Department of Plastic Surgery, Charing Cross Hospital, Imperial College Healthcare National Health Service Trust, London, UK; 3 Department of General Medicine, University Hospital Lewisham, University Hospital Lewisham National Health Service Trust, Lewisham, London, UK

## Abstract

Venous thromboembolism is a very common pathological process for which there are many well known (and less well-known) predisposing factors. Likewise, olanzapine is a commonly used anti-psychotic medication.

We present the case of a young Somali gentleman who developed venous thromboembolic disease after an overdose of olanzapine. The diagnosis was only made 48 hours after admission, due to the non-specific presentation of the pulmonary embolus and the fact that the link between olanzapine and pulmonary embolus was not previously widely described and therefore it did not immediately figure in the differential diagnosis. The patient made a full recovery.

There is an increasing body of circumstantial evidence linking olanzapine to pulmonary embolus. Clinicians should bear this possible association in mind when prescribing the drug and when faced with clinical situations where venous thromboembolism (VTE) is a possible diagnosis. VTE has occasionally been described in therapeutic dose olanzapine therapy, but never in the context of an acute overdose. Khat, a recreational drug, has been linked to arterial, but not venous thrombosis.

It is hoped that this case report will further encourage research into these associations, which remain to be proven and quantified.

In the context of changing population demographics and increasing global migration, a greater awareness of the potential effects of endemic practices and their potential consequences is essential to the modern-day doctor working in a multi-cultural society.

## Introduction

Olanzapine is an atypical anti-psychotic, very commonly used in the management of schizophrenia and mania. There are a number of reported side effects of this drug, including weight-gain, sedation and anti-muscarinic effects but until recently there was no known link to thromboembolic disease.

Venous thromboembolism is a common pathological process for which many risk factors have been described. The commonest are immobility, malignancy, surgery and hypercoagulable states. Some drugs, including the typical anti-psychotics, have long been known to increase the risk of venous thromboembolism, however it was not until very recently that atypical anti-psychotics and indeed olanzapine were shown to be related to this disorder.

We describe an interesting case of massive pulmonary embolism temporally related to ingestion of an olanzapine overdose and explore the existing literature on the topic.

## Case presentation

### Case Presentation

A 27-year old, obese Somali gentleman was admitted with a history of acute onset of hallucinations and delirious behaviour. He was found by his relatives with suspected olanzapine overdose and had been on the floor for more than two days. He had a past history of depression and psychosis for which he was on this medication. The patient also had a longstanding history of "Khat" abuse (a flowering plant with amphetamine-like effects). On admission, he was noted to be disorientated and confused. He was afebrile, tachycardic and had a blood pressure of 110/70. His oxygen saturations were normal. Cardiovascular, respiratory, abdominal and neurological examinations were unremarkable. There were no signs of meningism.

### Investigations

Blood tests revealed no significant abnormalities apart from elevated Creatine Kinase of 4,800 u/l, which was presumed to be due to rhabdomyolysis secondary to lying on the floor for a prolonged period. Arterial blood gases and chest X-RAY were normal, whilst his ECG showed sinus tachycardia. A CT scan of his brain did not reveal any abnormalities. Urine dipstick showed no evidence of a urinary tract infection. Serial ECGs showed the development of widespread flattened T waves and right axis deviation. A CT pulmonary angiogram was performed 48 hours after admission and revealed extensive pulmonary thrombo-embolic disease and right lower lobe consolidation.

### Differential Diagnosis

• Cardiac event

• Drug-induced arrhythmia

• Pulmonary embolus

• Lower respiratory tract infection

### Treatment

He was started on intravenous antibiotics for possible aspiration pneumonia and anticoagulation for pulmonary embolism.

### Outcome and Follow up

He was subsequently reviewed by the psychiatrists and admitted to storing 90 olanzapine tablets and taking these with a clear suicidal intent following a severe depressive episode. He denied having taken any other medications and no other empty packaging was found, apart from that of his olanzapine tablets. Following medical treatment for the pulmonary embolus and lower respiratory tract infection, he was discharged to the care of the mental health team. He has since recovered completely.

## Discussion

The link between conventional anti-psychotic medications and venous thromboembolism (VTE) was first suggested in the 1950s. However, there have been few case reports and case-control studies to support an association between atypical anti-psychotics and VTE and these are mainly related to clozapine. One retrospective study of nursing home residents suggested that atypical anti-psychotics appear to increase the risk of VTE, though such events remain rare [1]. It also showed that, of the atypical agents commonly used, clozapine had the strongest links to VTE [1]. One series described three elderly patients with possible association between chronic use of olanzapine and VTE and there is one case report of VTE in a young male after starting olanzapine therapy.

In recent months, three further reports detailing six more cases of VTE in patients on olanzapine were published, however in four of these cases, multiple other clinical and biochemical risk factors for VTE were present. A team of forensic pathologists from USA has reported six cases of fatal pulmonary embolus occurring over a seven year period where the patients concerned were on olanzapine therapy, which may therefore have been a contributing factor [2].

There have been few mechanisms postulated to explain these associations. Clozapine, but not the other atypical antipsychotics, has been shown to have a weak association with in-vitro platelet adhesion, aggregation and plasma coagulation [3]. Atypical anti-psychotics have been associated with sedating effects, a more sedentary lifestyle and weight gain, all of which are predisposing risk factors for VTE [4]. Indeed, this patient was obese and had been immobilised for two days prior to hospital admission, due to sedation induced by the overdose. He worked as a taxi driver and stated that he did no regular exercise.

Atypical agents also possess a high affinity for the serotonin receptor type 2A, and serotonin-induced platelet aggregation may be affected [5]. Metabolic abnormalities such as dyslipidaemia, hyperleptinaemia, hyperglycaemia and hyperhomocysteinaemia have been observed in users of atypical antipsychotics [4] and all are associated with decreased fibrinolytic activity. However, these are unlikely to be aetiologic factors in early thromboembolic occurrence [1].

Recent small and preliminary studies have yielded conflicting results, with one retrospective study of the WHO database for adverse drug reactions noting that pulmonary embolism occurred with a higher frequency in patients on olanzapine than on other drugs, though conceding that these conclusions were based on incomplete data [6]. Another study in 2008 concluded that olanzapine did not have any direct effect on pro- or anticoagulant activity or fibrinogen levels. Although this study showed that patients on long-term olanzapine therapy had increased leptin and PAI-1 levels compared to controls, they did not have significantly different levels to those in their untreated first degree relatives. The authors postulated that increased incidence of VTE in these individuals may be due to obesity and lifestyle rather than an effect on coagulation [7]. The link between VTE and this patient subset may also be due to other factors relating to lifestyle that are associated with these illnesses [4].

Khat (*Catha edulis Forsk*, also known as qat, mirra, jaad or qaad) is a herbal drug, widely used in East Africa and the Arabian Peninsula for centuries. The main active ingredient is cathinone, a sympathomimetic amine alkaloid thought to have effects similar to amphetamines [8] including euphoria, hyperexcitability, insomnia, paranoia and psychosis. Peripheral effects of khat may be related to release of norepinephrine, leading to arterial hypertension and an increased heart rate [9].

Potential links between Khat use and arterial thrombosis [9,10] (ischaemic stroke and myocardial infarct through coronary vasospasm, catecholamine-mediated platelet aggregation and increased myocardial oxygen demand) have been described in the literature, but to our knowledge none with VTE.

This gentleman's pulmonary embolus could have occurred at any point from ingestion of the olanzapine tablets and during his subsequent immobilisation, until his CTPA scan. The fact that he had a sinus tachycardia (the commonest ECG finding in pulmonary embolus) that did not respond to adequate fluid resuscitation would suggest that this was not simple dehydration. He developed dynamic ECG changes within 36 hours of admission and his saturations dropped, probably as the thromboembolic episode progressed.

His obesity and period of immobilisation predisposed to VTE and therapeutic doses of olanzapine have been linked to VTE. This case report poses the question of olanzapine overdose, khat abuse or the combination of both having contributed to his life-threatening thromboembolic episode.

## Conclusion

There appears to be a possible relationship between use of olanzapine and venous thromboembolism and further study into this is needed to confirm and quantify it. It remains to be seen whether this relationship is dose dependant, as might be suggested by the presentation in our case. Clinicians should bear this in mind when prescribing this drug and when faced with clinical situations where venous thromboembolism is an obvious (or not so obvious!) diagnosis. Although there is no evidence as yet, one might also exercise caution when prescribing olanzapine to patients at high risk of VTE. VTE has occasionally been described in therapeutic dose olanzapine therapy, but never in the context of an acute overdose. It is hoped that this case report will further encourage study of the relationship.

### Take Home Points

• There is an increasing body of circumstantial evidence linking olanzapine to venous thromboembolism.

• Pulmonary embolism should be considered in the differential diagnosis of patients presenting with non-specific features of this condition, even if they initially appear to have no risk factors.

• In the context of changing population demographics and increasing global migration, a greater awareness of the potential effects of endemic practices is essential.

## List Of Abbreviations

VTE: Venous thromboembolism; PAI-1: Plasminogen Activator Inhibitor-1

## Consent

Written informed consent was obtained from the patient for publication of this case report and accompanying images. This was also witnessed by the patient's father, who has also signed the consent form. A copy of the written consent is available for review by the Editor-in-Chief of this journal.

## Competing interests

The authors declare that they have no competing interests.

## Authors' contributions

JM wrote up the case report, case presentation, discussion and conclusions and reviewed the literature for similar cases and studies on the topics referred to in the discussion and conclusion. GD assisted with literature review. KN provided help with writing up the case presentation. MP provided guidance in the formulation of the case report from the data collected and the comparison of this case to those in the literature.

All authors have read and approved the final manuscript.
